# RNA Interference-Mediated Knockdown of Genes Encoding Spore Wall Proteins Confers Protection against *Nosema ceranae* Infection in the European Honey Bee, *Apis mellifera*

**DOI:** 10.3390/microorganisms9030505

**Published:** 2021-02-27

**Authors:** Nan He, Yi Zhang, Xin Le Duan, Jiang Hong Li, Wei-Fone Huang, Jay D. Evans, Gloria DeGrandi-Hoffman, Yan Ping Chen, Shao Kang Huang

**Affiliations:** 1 College of Animal Sciences (Bee Science), Fujian Agriculture and Forestry University, Fuzhou 350002, China; ywxkng@yeah.net (N.H.); xinleduan@fafu.edu.cn (X.L.D.); leejh6972@126.com (J.H.L.); wfhuang@fafu.edu.cn (W.-F.H.); 2 Guangdong Key Laboratory of Animal Conservation and Resource Utilization, Guangdong Public Laboratory of Wild Animal Conservation and Utilization, Institute of Zoology, Guangdong Academy of Sciences, Guanzhou 510260, China; zy3001@163.com; 3 U.S. Department of Agriculture-Agricultural Research Service Bee Research Laboratory, Beltsville, MD 20705, USA; jay.evans@usda.gov; 4 USDA-ARS Carl Hayden Bee Research Center, 2000 East Allen Road, Tucson, AZ 85719, USA; gloria.hoffman@usda.gov

**Keywords:** *Nosema ceranae*, *Apis mellifera*, RNA interference, spore wall proteins, silencing, spore load, immunity, survivor

## Abstract

*Nosema ceranae* (Opisthosporidia: Microsporidia) is an emergent intracellular parasite of the European honey bee (*Apis mellifera*) and causes serious *Nosema* disease which has been associated with worldwide honey bee colony losses. The only registered treatment for *Nosema* disease is fumagillin-b, and this has raised concerns about resistance and off-target effects. Fumagillin-B is banned from use in honey bee colonies in many countries, particularly in Europe. As a result, there is an urgent need for new and effective therapeutic options to treat *Nosema* disease in honey bees. An RNA interference (RNAi)-based approach can be a potent strategy for controlling diseases in honey bees. We explored the therapeutic potential of silencing the sequences of two *N. ceranae* encoded spore wall protein (SWP) genes by means of the RNAi-based methodology. Our study revealed that the oral ingestion of dsRNAs corresponding to SWP8 and SWP12 used separately or in combination could lead to a significant reduction in spore load, improve immunity, and extend the lifespan of *N. ceranae*-infected bees. The results from the work completed here enhance our understanding of honey bee host responses to microsporidia infection and highlight that RNAi-based therapeutics are a promising treatment for honey bee diseases.

## 1. Introduction

Honey bees are one of the most important pollinators in the agricultural and ecological system and are responsible for pollinating approximately 30% of all the food we consume [1,2]. The total economic value of pollination service by insects, which can mostly be credited to honey bees, amounted to EUR 153 (USD 187) billion worldwide in 2005; this represents 9.5% of the value of global agricultural production for human food [3]. Over the past few decades, however, there has been a serious decline in honey bee populations in many parts of the world, especially in the USA and some European countries, threatening the global food supply and worldwide environmental biodiversity [4,5,6,7].

The decline in bee populations has been attributed to various causes, including parasites and diseases, pesticide use, habitat loss, poor nutrition, climate change, and other environmental stressors that act in isolation or synergistically [8,9]. Among the multiple factors that negatively affect bee health, pathogens and parasites represent a major threat to the vitality of honey bees and the economic stability of the commercial beekeeping industry. The unicellular microsporidia *Nosema ceranae* is a parasite of the European honey bee [10,11], and has been responsible for worldwide colony losses since its first discovery in *Apis mellifera* in 1996 [12,13,14,15,16,17]. As is the case with other microsporidia that have mitochondrial remnants known as mitosomes and have recently been reclassified as fungi [18], *N. ceranae* produces highly infectious and resistant spores. Honey bees become infected with *Nosema* via a fecal-oral route when they ingest food or water contaminated with spores or clean spore-contaminated combs. Under suitable conditions in the midgut, the ingested spores germinate with a polar tubule extruding from a spore. The polar tubule pierces the membrane of the host’s midgut epithelial cell and serves as a conduit to deliver infectious sporoplasm into the host cell. The intracellular phase of the spore’s life-cycle consists of a proliferative phase (merogony), the spore production phase (sporogony), and the mature spore or infective phase [19]. The mature spores either infect adjacent cells or are released into the midgut lumen via cell lysis and are excreted in feces into the hive environment, thus providing new sources of infection. Through repeated multiplication, roughly 30 to 50 million *Nosema* spores accumulate inside a bee’s midgut within two weeks of the initial infection [20].

Honey bee health is affected by *Nosema* disease in multiple ways. *N. ceranae* predominantly multiplies in the midgut epithelium of honey bees. However, the damage done during infection is not limited to the digestive system. The overall physiology of infected bees is impacted, including suppression of immune function, inhibition of intestinal epithelial cell apoptosis [21,22], disturbance of hormone homeostasis [23], disruption of homing ability, lifespan reduction [24,25,26], and severe winter mortality [27,28]. Furthermore, *Nosema* infection renders bees more vulnerable to extreme temperatures, nutritional stress, and other bee pathogens [25,29,30,31]. New evidence shows that interactions between *N. ceranae* and neuro-active pesticides such as neonicotinoids could synergistically and negatively affect honey bee survival and significantly contribute to colony depopulation [32,33,34,35,36]. If the queen of the colony becomes infected, her egg-laying rate is reduced due to atrophy of the oocytes, and she is likely to be superseded by the workers. As a result, *Nosema*-infected colonies often become queenless, and bee populations can drop dramatically [37,38].

Nosemosis type C, a *Nosema* disease caused by *N. ceranae* [39], is transmitted among honey bees via environmentally resistant and infectious spores, which are one of the principal features of microsporidia. The resistant spores are protected by a thick and rigid wall consisting of two layers, the electron-dense outer layer (exospore) and the electron-lucent inner layer (endospore), which are separated from the cell by a thin plasma membrane and contain a polar tube that forms 18–24 coils along the inner wall of the spore [19]. Spore wall proteins are localized on the surfaces of mature spores. Over the past decade, characterizations of the composition and function of microsporidian spore walls have mainly focused on the study of the *Encephalitozoonidae* genus associated with human infections and *Nosema bombycis,* which is still the most serious pathogen of silkworms. Several spore wall proteins (SWPs) were identified in their exospore or endospore [40,41,42,43]. The spore wall proteins are thought to be the first proteins to directly interact with the host cell, playing a key role in host cell adherence and invasion in partnership with the invasion organelle, in the form of a polar tube, which is another principal feature of microsporidia. Genome sequencing and analyses of *N. ceranae* identified multiple genes encoding spore wall proteins (SWPs) [44,45]; however, their biological role in host cell invasion and pathogenesis remain to be characterized.

Among the numerous compounds that have been tested against *Nosema*, fumagillin is the only commercialized antibiotic for use against *Nosema*. The active ingredient in fumagillin was isolated from the fungus *Aspergillus fumigatus* and suppresses the reproduction of *Nosema* in honeybees. However, there are several problems associated with fumagillin [46,47,48,49,50]: First, fumagillin inhibits the reproduction of spores but does not kill them; therefore, treatment with this antibiotic does not completely eliminate nosemosis (*Nosema* disease) from the colony, and infection may return after chemical therapy is stopped. Second, *Nosema* parasites in honey bees have developed resistance to fumagillin. Finally, utilization of Fumagillin is prohibited in the European Union because it has no established maximum residue level (MRL), required for treatments employed in food-producing animals (FPA). As a result, additional therapeutic options are urgently needed for the treatment of *Nosema* infections in honey bees.

Plant-derived secondary metabolites, natural products, have been used for the treatment of various diseases in humans and animals due to their safety and a broad spectrum of bioactivities. Likewise, the effects of natural products on *N. ceranae* infections have been explored as alternatives to the antibiotic fumagillin [51]. Of the plant natural products explored to date, the extract of propolis, which is a mixture of resinous substances collected by honeybees from different plant sources and used by bees to defend the hive, has been shown to reduce *N. ceranae* spore load in infected bees and increase the survival of the European honey bee [52] and other bee species [53,54] following oral treatment. In addition, organic compounds such as porphyrins and nutraceutical and immuno-stimulatory compounds have been shown to exhibit antimicrobial properties and inhibit the infectivity of *N. ceranae* in honey bees [55,56,57], providing possible therapeutic modalities for the *Nosema* disease treatment.

RNA interference (RNAi) is an evolutionarily conserved mechanism that is triggered by double-stranded RNA (dsRNA) and knocks down gene expression at the translation stage in a range of organisms, including insects [58]. Whole-genome sequence analysis of *N. ceranae* and comparative genomic analysis of *N. ceranae* with its sympatric congener *N. apis* have led to the identification of various virulence genes [44,59] that are important for the microbial invasion and pathogenesis in honey bees. The genes are potential targets for innovative therapeutics to break down the life cycle of the parasite. For example, silencing virulence genes via oral ingestion of a dsRNA corresponding to the sequences of *N. ceranae* polar tube protein 3 (PTP3) and ADP/ATP transporter, which are involved in the host cell’s invasion and parasite replication within them, might suppress gene expression and reduce the *Nosema* load, thus extending the lifespan of infected bees [60,61]. In addition, a subsequent study showed that *N. ceranae* spore loads could be significantly reduced after feeding infected honeybees with RNAi that targets the *N. ceranae* gene coding Dicer (siRNA-Dicer), which broadly regulates *N. ceranae* proliferation and honey bee metabolism [62]. Moreover, a therapeutic approach targeting the host factors showed that, while the expression of a honey bee gene, naked cuticle (nkd), which is a negative regulator of host immune function, was induced by *N. ceranae* infection, the RNAi-mediated knockdown of nkd transcripts could efficiently silence nkd expression, reduce *N. ceranae* spore loads, and extend the lifespan of honey bees infected with *N. ceranae* [63]. All of these results strongly suggest that RNAi-based therapeutics hold great promise for the effective treatment of honey bee *Nosema* diseases and warrant further investigation.

To further explore the therapeutic potential of the RNAi-based strategy for controlling honey bee diseases, in the present study, we conducted a study to assess the effects of silencing the sequences of two *N. ceranae* encoded spore wall protein (SWP) genes on *N. ceranae* spore production and honey bee overall fitness. Our study revealed that oral ingestion of dsRNAs corresponding to SWP8 and SWP12 used separately or in combination could lead to a significant reduction in spore load and improve the overall fitness of *N. ceranae* infected bees. The results from our studies highlight that RNAi-based therapeutics can be an effective treatment of honey bee diseases and have positive implications for bee disease management.

## 2. Materials and Methods

### 2.1. Ethics Statement

The experimental honey bee colonies (*Apis mellifera ligustica*) were reared in the USDA-ARS Bee Research Laboratory apiaries, Beltsville, Maryland, USA. The apiaries are the property of the USDA-ARS and are not privately owned or protected in any way. No specific permits were required for the described studies. The studies involved the European honey bee (*Apis mellifera ligustica*), which is neither an endangered nor protected species.

### 2.2. Nosema ceranae Spore Purification

About three hundred honey bee adult workers were collected from colonies that were identified with a high level of *N. ceranae* infection (10^7^ spores/bee) [64,65]. After immobilizing bees by chilling with ice, the guts were removed from individual bees by pulling the stinger with forceps. The midguts were dissected out, pooled in a 5 mL plastic tube, and homogenized in water. The homogenate was centrifuged by differential centrifugation at 5000 g for five minutes to pellet the spores. The supernatant containing tissue and pollen debris was discarded. The spore pellet was washed in sterile water twice following the same centrifugation method described above. The concentration of spores was determined using a hemocytometer (Neubauer-ruled Bright Line counting chambers; Hausser Scientific, Horsham, PA, USA) under light microscopy [64] and diluted to 5 × 10^3^ spores/mL in 50% (w/v) sucrose solution. The spore solution was stored at 4 °C until use.

### 2.3. dsRNA Synthesis

The mRNA sequences of two *N. ceranae* spore wall proteins (SWP8, GenBank accession #: XM_024475065; SWP12, GenBank accession #: XM_024475810) were downloaded from the National Center for Biotechnology Information (NCBI)’s database. The primer pairs for dsRNA synthesis were designed from the mRNA sequences of SWP8 and SWP12 that share highly conserved spore wall protein sequences across different microsporidia isolates in GenBank and without matches to non-target genes using BLASTN searches. E-RNAi web service was used to design and evaluate long dsRNAs [66]. Primers were designed using the E-RNAi web tool [66]. Primers for the production of dsRNA of SWP8 and SWP12 were fused with a T7 promoter sequence (5′-taa tac gac tca cta tag ggc ga-3′) individually. Primers used in the study are listed in Table 1.

To obtain *N. ceranae* DNA, 50 μL of the spore solution was disrupted for 45 s at 65 m/s speed using a FastPrep cell disrupter (Thermo Labsystems INC, Philadelphia, PA, USA) in a 2.0 mL tube containing sterile 1.4 mm zirconium silicate grinding beads (Chemco, Montoursville, PA USA) and 400 µl CTAB buffer (100 mM Tris-HCl, pH 8.0, 20 mM EDTA, pH 8.0, 1.4 M sodium chloride, 2% cetyltrimethylammonium bromide, 0.2% 2-mercaptoethanol) with proteinase K (200 µg/mL). The mixture was then incubated overnight at 50 °C. One milliliter of DNAzol reagent (Thermo Fisher Scientific, Waltham, MA, USA), a ready-to-use organic reagent for the isolation of genomic DNA, was added and mixed using the FastPrep disrupter again. The homogenate was centrifuged for 10 min at 10,000× *g*. The supernatant was transferred to a new tube with 500 µl of ethanol absolute and incubated for at least 30 min at −20 °C. The suspension was centrifuged for 30 min at 10,000× *g*, and the pellet was washed with 70% ethanol. The resultant DNA pellet was resuspended in sterile water and stored at −20 °C until used.

A polymerase chain reaction (PCR) was performed on the DNA sample extracted from *N. ceranae* spores using SWP8F/SWP8R and SWP12F/SWP12R primer pairs which were tagged with T7 promoter sequence individually at 5′ ends (Table 1) to obtain DNA templates. The 100 μL PCR reaction mixture contained the following components: 78 μL H_2_O, 10 μL 10× reaction buffer, 3 μL MgCl2, 2 μL dNTP mix, 2 μL forward primer (20 μM), 2 μL reverse primer (20 μM), 1 μL Taq polymerase, and 2 μLDNA template. The thermal profile of the PCR amplification was as follows: one cycle of 94 °C for 3 min followed by 35 cycles of 94 °C for 30 s, 56 °C for 30 s and 72 °C for 90 s with a final extension of 72 °C for 10 min. After PCR amplification, gel electrophoresis in 1.5% low melt agarose gels was performed to verify the size of expected targets. PCR products were purified individually using Wizard PCR Prep DNA Purification System (Promega, Madison, WI) and sequenced. The sequences were Basic Local Alignment Search Tool (BLAST) searched to confirm the sequencing specificity.

The purified PCR products were used as templates for the in vitro transcription reaction. The dsRNA was synthesized using the MEGAscript^®^ RNAi Kit (Thermo Fisher Scientific, Waltham, MA, USA) following the manufacturer’s instructions. Briefly, the transcription reactions were assembled, and the incubation time was extended to 15 h at 37 °C. Nuclease digestion, purification, and elution followed using the materials supplied in the kit. The products of dsRNA were verified in 1.0% agarose gels and the concentration of dsRNA was determined with a spectrophotometer (NanoDrop 8000, Thermo^®^ Fisher Scientific, Waltham, MA, USA). The dose-dependent assay was performed to determine the optimized concentration of dsRNAs. The 1.5–1.8 μg/mL dsRNAs demonstrated the greatest efficacy with respect to spore count reduction, and therefore the concentration of 1.8 μg/mL dsRNAs was used for the subsequent bioassay.

### 2.4. RNAi Feeding Bioassays

Combs of sealed brood from colonies that were identified as *N. ceranae*-negative by monthly disease survey [64] were collected and placed in a mesh-walled cage and stored overnight in an insect growth chamber at 34 ± 1 °C, 55 ± 5% RH. Newly emerged bees were mixed in a plastic bin to avoid colony effects and allowed to roam on comb for 24–48 h to feed on stored pollen and honey in order to acquire gut microbiota. The bees then were removed from the frames and kept without food for at least 2 h before the subsequent *N. ceranae* inoculation. Individual bees were fed with 4 μL of inoculum solution (5 × 10^3^ spores/bee) via a P10 pipette. Uninfected bees (negative control) were fed with 4 μL of 50% sucrose syrup. The inoculated and control bees were transferred separately to the rearing cages [69,70]. The caged bees under controlled laboratory conditions were divided into five groups based on the treatment they received: Group I, contained untreated *N. ceranae*-infected bees; Group II. *N. ceranae*-infected bees + GFP-dsRNA; Group III, *N. ceranae* -infected bees + SWP8-dsRNA; Group IV, *N. ceranae* -infected bees + SWP12-dsRNA; and Group V, *N. ceranae* -infected bees + SWP8-dsRNA and SWP12-dsRNA. A group of bees without *N. ceranae* infection was used as a negative control and designated as Group 0. Bees in Groups 2–5 were fed dsRNA in a 50% sucrose solution *ad libitum* for the first six days of the study. The bees in single dsRNA treatment groups were fed with the solution containing 1.8 μg SWP8-dsRNA or SWP12-dsRNA/mL. The bees in dual dsRNAs treatment were fed with the solution containing 0.9 μg SWP8-dsRNA/mL and 0.9 ug SWP12-dsRNA/mL. Groups 0 and 1were fed with only 50% sugar solution. A piece of bee breadwas supplied at the bottom of each cage to provide proteins, lipids, and sterols. The sucrose solution was replaced every two days and the bee bread was changed every three days. All cages were maintained in an insect incubator (32 °C, 75% RH). Each experimental group consisted of six cages (35 bees/cage), three cages for molecular analysis and three for observation on bee survivorship and spore count. For cages used for molecular analysis, fifteen bees were sampled from each cage at day 12 and 15, respectively. For cages used for observation on bee survivorship and spore count, the number of dead bees was counted and removed daily. On day 20 after treatment, the observation of survivorship was completed and the midguts of survivor bees were removed and Nosema spores were counted.

### 2.5. Data Analysis

Relative expression of (1) SWP8, (2) SWP12, (3) *N. ceranae* 16S rRNA, (4) a gene encoding antimicrobial peptide, *Apidaecin*, and (5) a gene encoding signal-transducer and activator of tran-scription protein at 92E (Stat92E), a transcription factor of the JAK-STAT pathway in different experimental groups was interpreted by using the comparative Ct method (ΔΔCt Method) [71]. Briefly, the mean value and standard deviations of each target gene were normalized using the Ct value corresponding to the β-actin and the normalized value was expressed as ΔCt. The experimental group that had the lowest value was chosen as a calibrator and was equaled to 1. The ΔCt value of each group was subtracted by ΔCt value of the calibrator to yield ΔΔCt. The relative expression of each target gene among different experimental treatment groups was expressed as fold-difference in the following equation: 2^−ΔΔCt^.

After confirmation of a normal distribution and an equal variance of data, a one-way ANOVA was used to determine whether treatment groups differed in the expression of each target gene. *N. ceranae* spore load was compared among treatment groups using ANOVA. Tukey’s Honestly Significant Difference (HSD) test was used for all pairwise comparisons among sample means, survival analysis was performed using the Gehan–Breslow–Wilcoxon test, and log-rank tests were used to assess the impact of dsRNA treatment on the improvement on the survivorship of Nosema-infected bees with single or dual dsRNA treatments in comparison to Nosema-infected bees without treatment as well as to healthy bees. All analyses were carried out and all the figures were generated using Graphpad Prism 8 (GraphPad Software, Inc., San Diego, CA, USA).

## 3. Results

### 3.1. Ingestion of dsRNAs Corresponding to the Sequences of N. ceranae SWP8 Separately or in Combination with SWP12 Suppresses the Gene Expression SWP8 in Infected Bees

**Figure 1:**
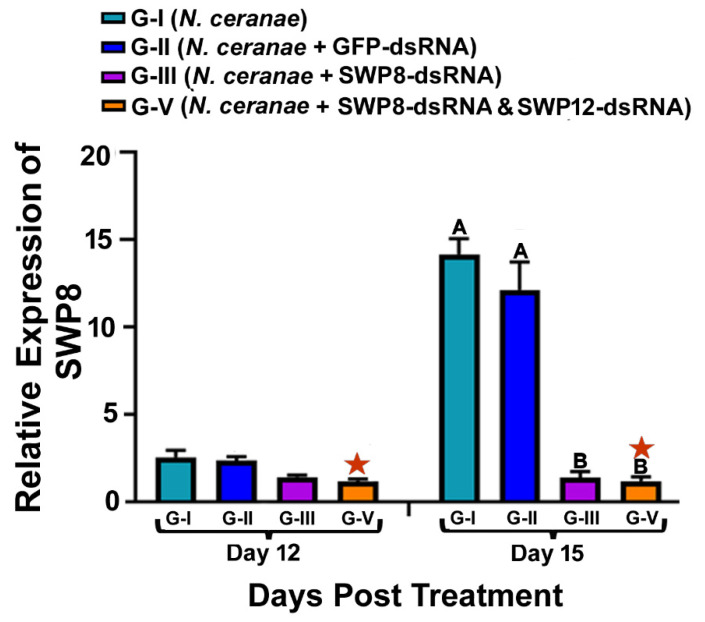
Relative expression of SWP8 in different treatment groups of honey bees. The X-axis indicates different experiment groups at day 12 and day 15 post treatment. The Y-axis depicts the fold difference in the relative expression of SWP8 relative to the calibrator, which is marked by a star and has the lowest expression level and is equal to one. The different uppercase letters above the bars indicate the statistically significant difference (*p* ≤ 0.05, one-way ANOVA and Tukey’s Honestly Significant Difference (HSD) tests) among the various groups on day 12 after treatment; meanwhile (*p* ≤ 0.05, one-way ANOVA and Tukey’s tests) among different groups on day 15 post-treatment.

As reflected in Figure 1, at day 15 post treatment, there was a significant difference in the relative expression of SWP8 among different treatment groups and the silencing effect of SWP8-dsRNA in suppressing transcript level of SWP8 was significant (one-way ANOVA and Tukey’s HSD test: F(3,8) = 54.94, *p* < 0.0001,; G-I vs. G-III, *p* < 0.0001;G-II vs. G-III, *p* = 0.0002); approximately the same level of suppression in SWP8 expression was also observed in the bees in Group V, which received a treatment where SWP8-dsRNA and SWP12-dsRNA were mixed (day 12, G-I vs. G-V, *p* = 0.0060, G-II vs. G-V, *p* = 0.0027; day 15, G-I vs. G-V, *p* < 0.0001, G-II vs. G-V, *p* = 0.0002). However, there was no apparent additive or synergistic effect of the mixture of SWP8-dsRNA and SWP12-dsRNA in terms of suppressing SWP8 gene expression when compared to SWP8-dsRNA being administered alone (day 12, G-III vs. G-V, *p* = 0.6873; day 15, G-III vs. G-V, *p* > 0.9999). GFP-dsRNA did not trigger RNAi responses and there was no significant difference in the SWP8 gene expression between Group I (*N. ceranae* only) and Group II (*N. ceranae* + GFP-dsRNA) by day 12 and 15 after treatment (day 12: G-I vs. G-II, *p* = 0.9528; day 15: G-I vs. G-II, *p* =0.4693), excluding the off-target effects of the GFP-dsRNA. (Figure 1).

### 3.2. Ingestion of dsRNAs Corresponding to the Sequences of N. ceranae SWP12 Alone or in Combination with SWP8 Suppresses the Gene Expression of SWP12 in Infected Bees

**Figure 2:**
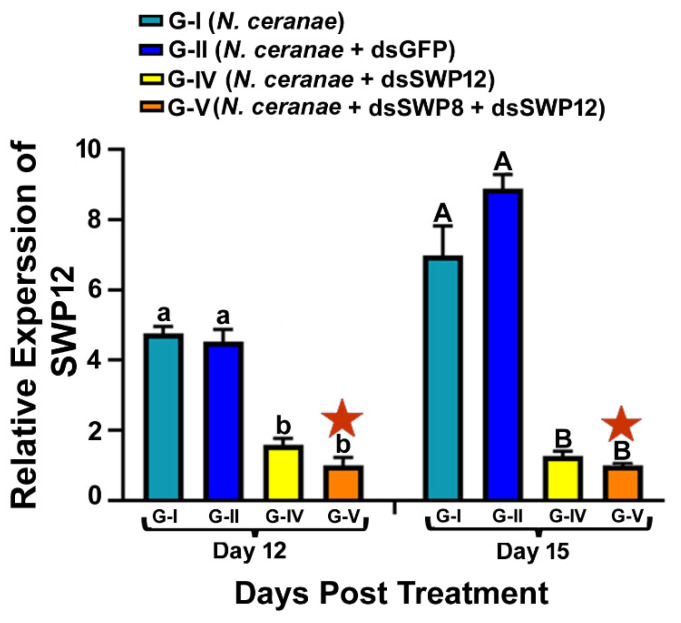
Relative expression of SWP12 in different treatment groups of honey bees. The X-axis indicates different experiment groups at day 12 and day 15 post treatment. The Y-axis depicts the fold difference in the relative expression of SWP12 relative to the calibrator, which is marked by a star and has the lowest expression level and is equal to one. The different lowercase letters above the bars indicate the statistically significant difference (*p* ≤ 0.05, one-way ANOVA and Tukey’s HSD tests) among the various groups on day 12 after treatment; meanwhile, the different uppercase letters above the bars indicate the statistically significant difference (*p* ≤ 0.05, One-way ANOVA and Tukey’s HSD tests) among different groups on day 15 post-treatment.

The ingestion of dsRNAs corresponding to the sequences of *N. ceranae* SWP12 elicited an earlier silencing response in *N. ceranae*-infected bees in comparison with SWP8-dsRNA. There was a significant difference in the relative expression of SWP12 among different experimental groups at both day 12 and 15 post treatment, and the mRNA transcript level of the SWP12 in Group IV (SWP12-dsRNA) and in Group V (SWP8-dsRNA and SWP12-dsRNA) was significantly reduced at day 12 and day 15 post treatment when compared to Group I (one-way ANOVA and Tukey’s HSD test. Day 12, F (3,8) = 58.73, *p* < 0.0001.G-I vs. G-IV, *p* = 0.0001; G-I vs. G-V, *p* < 0.0001, G-II vs. G-IV, *p* = 0.0002; G-II vs. G-V, *p* < 0.0001). (one-way ANOVA and Tukey’s HSD test. Day 15, F (3, 8) = 71.65, *p* < 0.0001; GI vs. G-IV, *p* = 0.0001; G-I vs. G-V, *p* < 0.0001; G-II vs. G-IV, *p* = 0.0212; G-II vs. G-V, *p* = 0.0006). The mRNA transcript level of the SWP12 in Group V bees was slightly lower than for bees in Group IV where SWP12-dsRNA was administered alone, but the difference was not significant (day 12: G-IV vs. G-V, *p* = 0.4062; day 15: G-IV vs. G-V, *p* = 0.9755). Additionally, there was no difference in the SWP12 gene expression between Group I (*N. ceranae* only) and Group II (*N. ceranae* + GFP-dsRNA) at day 12 and day 15 post treatment (day 12: G-I vs. G-II, *p* =0.9110; day 15: G-I vs. G-II, *p* = 0.0815) (Figure 2).

### 3.3. Silencing of the SWP8 and/or SWP12 Transcript Levels Led to the Significant Reduction in N. ceranae 16S rRNA Level and the Spore Load

**Figure 3:**
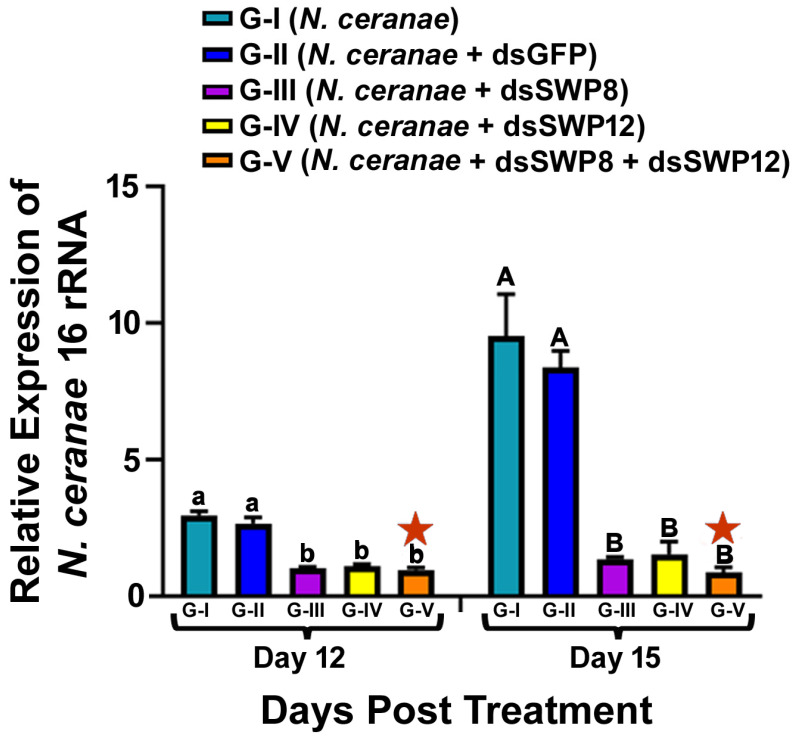
Relative expression of *N. ceranae* 16S rRNA in different treatment groups of honey bees. The X-axis indicates different experiment groups at day 12 and day 15 post treatment. The Y-axis depicts the fold difference in the relative expression of *N. ceranae* 16S rRNA relative to the calibrator, which is marked by a star and has the lowest expression level and is equal to one. The different lowercase letters above the bars indicate the statistically significant difference (*p* ≤ 0.05, one-way ANOVA and Tukey’s HSD tests) among the various groups on day 12 after treatment; meanwhile, the different uppercase letters above the bars indicate the statistically significant difference (*p* ≤ 0.05, One-way ANOVA and Tukey’s HSD tests) among different groups on day 15 post-treatment.

Ingestion of SWP8-dsRNA and/or SWP12-dsRNA knocked down the SWP8 or/and SWP12 mRNA transcript level, which in turn led to a significant reduction in *N. ceranae* levels. Analysis of *N. ceranae* 16S rRNA levels after dsRNA treatment showed that the amount of *N. ceranae* 16S rRNA in bees treated with SWP8-dsRNA, SWP12-dsRNA, or SWP8-dsRNA&SWP12-dsRNA (Group III, IV, and V, respectively), indicated significantly lower Nosema spore levels than in bees without treatment (Group I -*N. ceranae*) or treated with GFP-dsRNA (Group II)) at day 12 and day 15 after treatment (one-way ANOVA and Tukey’s HSD test. Day 12, F(4,10) = 51.23, *p* < 0.0001;G-I vs. G-II, *p* = 0.5456; G-I vs. G-III, *p* < 0.0001; G-I vs. G-IV, *p* < 0.0001; G-I vs. G-V, *p* < 0.0001; G-II vs. G-III, *p* < 0.0001; G-II vs. G-IV, *p* = 0.0007; G-II vs. G-V, *p* = 0.0003), (one-way ANOVA and Tukey’s HSD test. Day 15, F(4,10) = 30.56, *p* < 0.0001; G-I vs. G-II, *p* = 0.8195; G-I vs. G-III, *p* =0.0002; G-I vs. G-IV, *p* = 0.0002; G-I vs. G-V, *p* <0.0001; G-II vs. G-III, *p* =0.0005; G-II vs. G-IV, *p* = 0.0007; G-II vs. G-V, *p* = 0.0003). There was no significant difference in RNAi efficacy in inhibiting *N. ceranae* 16S rRNA expression between the single dsRNA (SWP8 or SWP12) and the mix of the two dsRNAs (day 12, G-III vs. G-V, *p* = 0.9948; G-IV vs. G-V, *p* = 0.9536; day 15, G-III vs. G-V, *p* = 0.9909; G-IV vs. G-V, *p* = 0.9714) (Figure 3).

**Figure 4:**
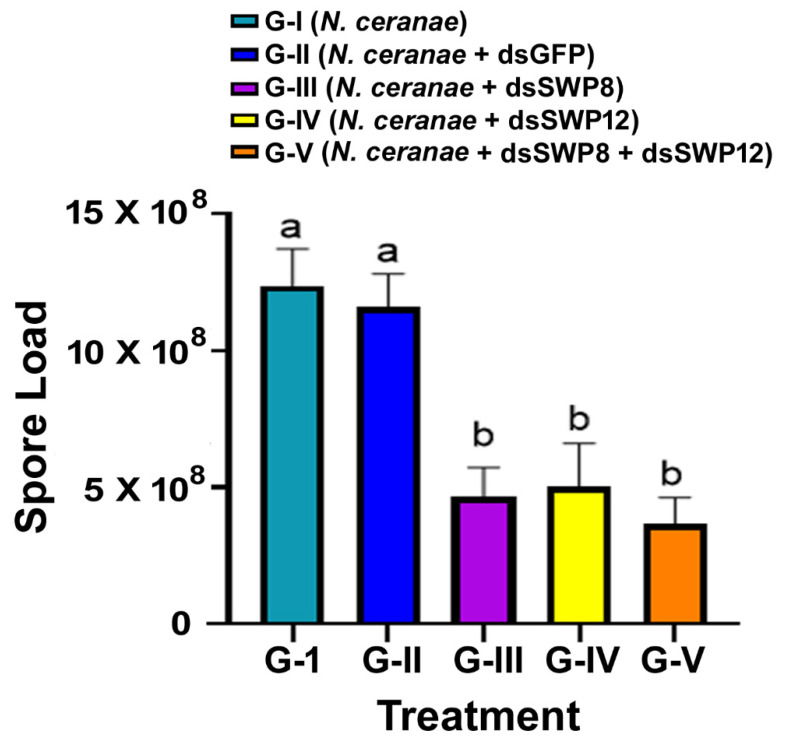
RNAi efficacy in silencing *Nosema* infection levels in infected honey bees. The *N. ceranae* infection levels were determined by spore counting. The X-axis indicates different treatment groups, while the Y-axis depicts spore loads, which are expressed as the mean ± SE. The different lowercase letters above the bars indicate the statistically significant difference (*p* ≤ 0.05, one-way ANOVA and Tukey’s HSD tests) among the various groups on day 20 after treatment.

Moreover, the reduction in *N. ceranae* 16S rRNA’s expression level occurred concurrently with the decrease in the *N. ceranae* spore load. There was a significant difference in the spore load among different experimental groups. The spore loads in Group III, IV, and V that were respectively treated with SWP8-dsRNA, SWP12-dsRNA, and SWP8-dsRNA&SWP12-dsRNA were significantly lower than among bees in Group I (*N. ceranae*) and Group II (*N. ceranae* + GFP-dsRNA) by day 20 post treatment (one-way ANOVA and Tukey’s HSD test. F(4,42) = 11.65, *p* < 0.0001; G-I vs. G-III, *p* = 0.0004; G-I vs. G-IV, *p* = 0.0038, G-I vs. G-V, *p* < 0.0001; G-II vs. G-III, *p* = 0.0042; G-II vs. G-IV, *p* = 0.0212; G-II vs. G-V, *p* = 0.0006). There was no difference in RNAi efficacy in relation to inhibiting spore load between the single dsRNA (SWP8 or SWP12) and the mix of the two dsRNAs (G-III vs. G-V, *p* = 0.9816; G-IV vs. G-V, *p* = 0.9595) (Figure 4).

### 3.4. Effects of SWP8 and/or SWP12 Silencing on Apidaecin Expression

**Figure 5:**
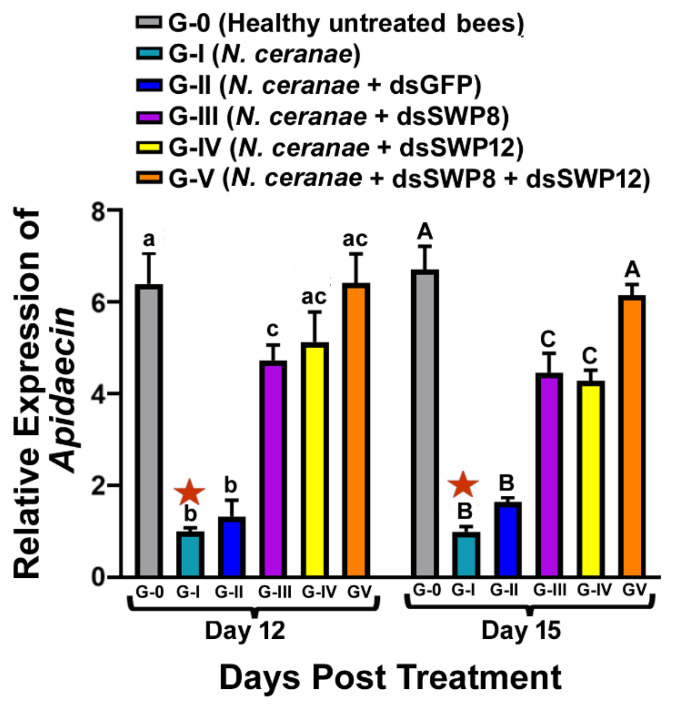
Relative expression of a gene encoding *apidaecin*, which is an immune effector of the Toll signaling pathway in different treatment groups of honey bees. The X-axis indicates different experiment groups at day 12 and day 15 post treatment. The Y-axis depicts the fold difference in the relative gene expression of *apidaecin* relative to the calibrator, which is marked by a star and has the lowest expression level and is equal to one. The different lowercase letters above the bars indicate the statistically significant difference (*p* ≤ 0.05, one-way ANOVA and Tukey’s HSD tests) among the various groups on day 12 after treatment; meanwhile, the different uppercase letters above the bars indicate the statistically significant difference (*p* ≤ 0.05, One-way ANOVA and Tukey’s HSD tests) among different groups on day 15 post-treatment.

The expression of a gene encoding *apidaecin*, an immune effector of the Toll signaling pathway, was used to measure RNAi efficacy of SWP8-dsRNA, and/or SWP12-dsRNA in modulating the immune response of *N. ceranae* infected bees. Expression analysis showed that *N. ceranae* infection could result in significant suppression of *apidaecin* gene expression in bees, and there was a significant difference in the relative expression of *apidaecin* among different experimental groups (one-way ANOVA and Tukey’s HSD test. Day 12: F (5,12) = 23.30, *p* < 0.0001; G-0 vs. G-I, *p* <0.0001) (one-way ANOVA and Tukey’s HSD test. Day 15, F (5,12) = 57.07, *p* < 0.0001; G-0 vs. G-I, *p* < 0.0001). This phenomenon in *N. ceranae* infected bees was found to be significantly relieved after knocking down the expression of SWP8 or SWP12 at day 12 and 15 post treatment (day 12: G-I vs. G-III, *p* = 0.0001; G-I vs. G-IV, *p* < 0.0001; G-I vs. G-V, *p* < 0.000; day 15: G-I vs. G-III, *p* < 0.0001; G-I vs. G-IV, *p* < 0.0001; G-I vs. G-V, *p* < 0.0001). Furthermore, significantly higher *apidaecin* mRNA expression occurred in bees fed a mix of two dsRNAs (Group V) compared with those fed a single dsRNA (Group III and Group IV) at both day 12 and 15 post treatment (day 12: G-III vs. G-V, *p* = 0.1206; G-IV vs. G-V, *p* = 0.4468, G-II vs. G-III, *p* = 0.0045; G-II vs. G-IV, *p* = 0.0018; G-II vs. G-V, *p* = 0.0001. Day 15: G-III vs. G-V, *p* = 0.1215; G-IV vs. G-V, *p* = 0.0598, G-II vs. G-III, *p* = 0.0003; G-II vs. G-IV, *p* = 0.0006; G-II vs. G-V, *p* < 0.0001.). There was no difference in *apidaecin* gene expression between Group I (*N. ceranae* only) and Group II (*N. ceranae* + GFP-dsRNA) at day 2 and day 15 post treatment (day 12: G-I vs. G-II, *p* > 0.9999; day 15: G-I vs. G-II, *p* = 0.9934) (Figure 5).

### 3.5. Effects of SWP8 and SWP12 Silencing on STAT92E Expression

STAT92E is a core component of the JAK-STAT signaling pathway. The expression of STAT82E in *N. ceranae*-infected bees (Group I) was markedly increased compared to uninfected bees (Group 0), and there was a significant difference in the relative expression of STAT92E among different experimental groups at day 12 post-treatment (one-way ANOVA and Tukey’s HSD test. Day 12, F(5,12) = 8.153, *p* < 0.0001; G-0 vs. G-I, *p* = 0.0174).

**Figure 6:**
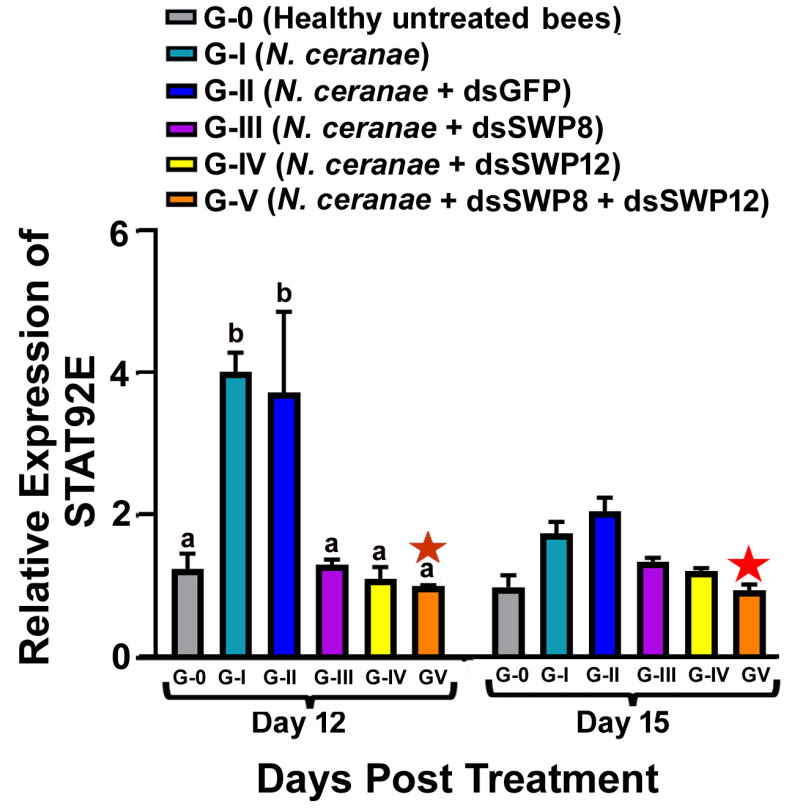
Relative expression of STAT92E, a core component of the JAK-STAT signalling pathway, in different experimental groups of honey bees. The X-axis indicates different experiment groups at day 12 and day 15 post dsRNA treatment. The Y-axis depicts the fold difference in the relative expression of STAT92E relative to the calibrator, which is marked by a star and has the lowest expression level and is equal to one. The different lowercase letters above the bars indicate the statistically significant difference (*p* ≤ 0.05, one-way ANOVA and Tukey’s HSD tests) among the various groups on day 12 after treatment.

When the expression of *N. ceranae* SWP8 and/or SWP12 genes was silenced, the expression of STAT92E was also reduced (day 12, G-I vs. G-III, *p* = 0.02; G-I vs. G-IV, *p* = 0.0123; G-I vs. G-V, *p* = 0.0098, G-II vs. G-III, *p* = 0.0408; G-II vs. G-IV, *p* = 0.0251; G-II vs. G-V, *p* = 0.0198. Day 15, G-I vs. G-III, *p* = 0.3497; G-I vs. G-IV, *p* = 0.1200; G-I vs. G-V, *p* = 0.0113, G-II vs. G-III, *p* = 0.0246; G-II vs. G-IV, *p* = 0.0073; G-II vs. G-V, *p* = 0.0098). There was no significant difference in RNAi efficacy between the mix of two dsRNAs and single dsRNA (SWP8 or SWP12) (day 12, G-III vs. G-V, *p* = 0.9977; G-IV vs. G-V, *p* > 0.9999. Day 15, G-III vs. G-V, *p* = 0.3274; G-IV vs. G-V, *p* = 0.7166). Additionally, there was no difference in the STAT92E gene expression between Group I (*N. ceranae* only) and Group II (N. ceranae + GFP-dsRNA) at day 12 and 15 post treatment (day 12: G-I vs. G-II, *p* = 0.9978; day 15: G-I vs. G-II, *p* = 0.5665) (Figure 6).

### 3.6. Silencing of SWP8 and SWP12 Gene Expression Contributed to Improved Survivorship

**Figure 7:**
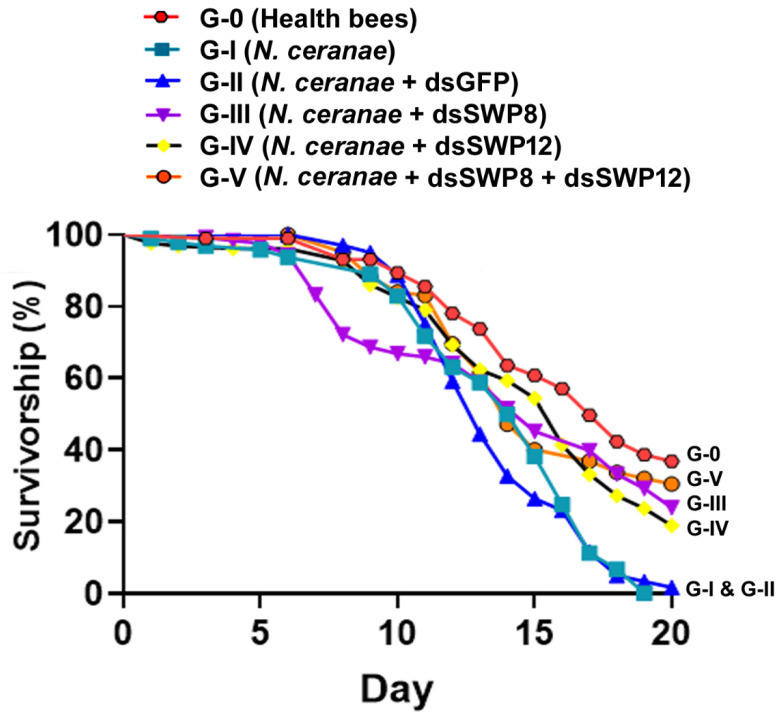
Effect of silencing SWP8 and SWP12 gene expression on the survivorship of adult workers. The X-axis indicates the days post treatment, while the Y-axis represents the survival rate (%) based on the daily accumulated mortality. Significant differences between the different groups were analyzed using the Kaplan–Meier Gehan–Breslow–Wilcoxon method. In addition, log-rank was employed to assess the overall homogeneity between the treatments. In all cases, *p* ≤ 0.05 was considered to be statistically significant.

The effect of dsRNA treatments on the survival of honey bees was assessed by measuring cumulative mortality in different experimental groups over a twenty-day observation period. The percent survival among six experimental groups was significantly different on day 20 after treatment (Gehan–Breslow–Wilcoxon (df = 5, *p* = 0.0019) and log-rank (df = 5, *p* < 0.0001) tests were performed). *N. ceranae*-infected bees in Group III, IV, and V that received SWP8-dsRNA, SWP12-dsRNA, and SWP8-dsRNA&SWP12-dsRNA treatment, respectively, had significantly greater survival rates than bees in Group I and II (Figure 7).

## 4. Discussion

RNAi is a major innate immune pathway of insects. Induced RNAi responses after feeding dsRNA to protect beneficial insects and control pests has been reported in various species that span different insect orders (reviewed in [72]. Complete genome analysis showed that the honey bee genome encodes all the key components of the RNAi machinery, including Dicer-like (DCL), Argonaute (AGO), and RNA-dependent RNA polymerase (RdRP) [73,74,75]. As such, RNAi has become a widely used tool specifically for the control of disease infections in honey bees. Furthermore, numerous studies have demonstrated the role of RNAi as a novel therapeutic strategy against various honey bee diseases caused by viruses, bacteria, fungi and parasitic *Varroa* mites [60,61,63,76,77,78,79,80,81,82,83].

In this study, we explored the therapeutic potential of silencing the expression of two *N. ceranae* encoded SWP genes, SWP8 and SWP12, by means of the RNAi-based strategy. We showed that reducing transcript levels of SWP8 and/or SWP12 could reduce the risk of *Nosema* disease, as shown by the fact that the number of spores in the infected bees receiving dsRNA treatment was significantly lower than in infected bees that went without treatment. While two sequences of SWP8 and SWP12 share a low degree of similarity at the amino acid level, dsRNA corresponding to the sequences of SWP8 and/or SWP12 was found to be highly effective at inducing RNAi response in *N. cerenae*-infected bees, although a significantly synergistic effect was not noted. Moreover, a reduction in the *N. ceranae* spore load in turn resulted in an overall enhanced lifespan in the infected bees, providing evidence of the involvement of SWPs in the *N. ceranae* sporogenesis and disease pathogenesis. This research underlines the potential of silencing *N. ceranae* virulence factors using RNAi as an effective way of controlling *Nosema* disease in honey bees.

Previous studies reported that *N. ceranae* infection could comprehensively and persistently suppress the immune system of honey bee, which in turn increases the susceptibility of the *N. ceranae* infected hosts to other bee pathogens and senescence [21,61,84,85,86,87]. As the first line of defense against invading pathogens, the insects’ innate immune system consists of a hemocyte-mediated cellular response and its humoral counterpart [87]. Humoral response refers to the activation of downstream intracellular signaling and the production of soluble effector molecules, known as antimicrobial peptides (AMPs), which are secreted into the hemolymph to control infections caused by invaders. The transcription of genes encoding for AMPs is regulated by several signaling cascade pathways such as Toll, Janus kinase-signal transducer and activator of transcription (JAK-STAT), and immune deficiency (Imd) pathways, which have all been described in the honey bee [88,89]. During pathogenic infections, the rapid production of AMPs as a part of the host’s innate immune defense is vital for preventing pathogens interacting with host cells and promoting pathogen clearance. Our study demonstrated that *N. ceranae*-infected bees (Groups I and II) had a notably lower expression level of the gene encoding immune peptide *apidaecin*, when compared with uninfected bees in Group 0. This result agrees with previous studies that show that *N. ceranae* infection significantly suppresses the honey bees’ immune response [61,63]. The immunosuppressive mechanisms that take place during *N. ceranae* infection may be one of the evolutionary driving forces for the widespread establishment of *N. ceranae* in honey bee host populations worldwide.

An interesting aspect of the RNAi response is that dsRNA treatment might not only result in a knockdown of specific gene expression post transcriptionally, but it may also regulate a signal transduction cascade, which has been implicated in host biological processes including immune responses. During our study, the suppression of hosts’ innate immune responses to *N. ceranae* infection in terms of *apidaecin* mRNA expression was found to be significantly reduced after knocking down the expression of SWP8 and/or SWP12. Furthermore, the elevation of *apidaecin* expression was significantly increased in bees fed with a mix of two dsRNAs compared with bees that received a single dsRNA, suggesting there is a synergistic effect between SWP8 and SWP12 that improves the immune response of *N. ceranae* infected bees.

At the same time, the Janus kinase protein (JAK) and the Signal transducer and activator of transcription (STAT) are two core components of the JAK-STAT signal pathway that plays critical roles in orchestrating this immune system and is an integral part of the immune response in insects including honey bees [88,89,90]. Seven STATs have been identified in humans, yet *Drosophila* STAT92E was the only STAT gene locus identified in insects. STAT92E encodes a transcription factor that shuttles between the cytosol and nucleus upon immune challenge and plays a critical role in cell growth, differentiation, and the immune response [91]. A previous study showed that shrimp STAT could be employed by the white spot syndrome virus (WSSV) as a transcription factor to enhance viral gene expression in host cells [92]. In our investigation, the expression of STAT82E in *N. ceranae*-infected bees (Group I) was markedly increased in comparison with uninfected and healthy bees (Group 0) at day 12 post treatment, implying that *N. ceranae* could manipulate the host transcription factor STAT82E. When the gene expression of *N. ceranae* virulent factors SWP8 and/or SWP12 was silenced, the expression of STAT92E was significantly reduced, demonstrating the role of RNAi response in regulating the signal transduction cascades involved in the host’s biological processes and immune responses. Additional research will determine the various roles linked to the activation of the JAK-STAT signaling pathway in complex host–pathogen interactions in honey bees.

In summary, the functional characterization of two *N. ceranae* SWPs in the present study indicates that SWP8 and SWP12 are good candidates for the development of the diagnosis and treatment of *Nosema* disease in honey bees. The results from this study, together with previous studies, confirm that RNAi-based therapeutics hold great promise for controlling disease infection in honey bees and improving their health.

## Figures and Tables

**Table 1:** Primer sequences used in this study.

Purpose	Gene Name	Primer	Sequence (5′---3′)	Reference	Length (bp)
dsRNA	*SWP8*	SWP8-F	taatacgactcactatagggagaCCATCAGTCATAACTTTGCC	This study	347
	*(N. ceranae)*	SWP8-R	taatacgactcactatagggagaTCTGCAAACTCTCCAACAAC		
dsRNA	*SWP12*	SWP12-F	taatacgactcactatagggagaAGGTAGGTTCAAGTATAGCC	This study	467
	*(N. ceranae)*	SWP12-R	taatacgactcactatagggagaCTAGTGCTTCTAGGCCAACT		
dsRNA	*GFP*	SWP12-F	taatacgactcactatagggagaATTCCATGGCCAACACTTGTCC	[67]	502
		SWP12-R	taatacgactcactatagggagaTCAAGAAGGACCATGTGGTC		
qPCR	*SWP8*	SWP8-qF	ACAAGTACTGCAGCAAATATAGGTT	This study	114
	*(N. ceranae)*	SWP8-qR	AATTGGCAAAGTTATGACTGATGGA		
qPCR	*SWP12*	SWP12-qF	AGTCAGAAGAATTGAATACAAGCAT	This study	148
	*(N. ceranae)*	SWP12-qR	CTTTGCATTACCCCCATGTTCA		
qPCR	*16S rRNA*	*16S rRNA*-qF	CGGATAAAAGAGTCCGTTACC	[14]	250
	*(N. ceranae)*	*16S rRNA*-qR	TGAGCAGGGTTCTAGGGAT		
qPCR	*STAT92E*	STAT92E-qF	TGAACCTGGAAGAGTGCCAT	This study	218
	*(A.mellifera)*	STAT92E-qR	TCTTGTCCGCTTGCATTTCC		
qPCR	*Apidaecin*	Apidaecin-qF	TTTTGCCTTAGCAATTCTTGTTG	[63]	80
	*(A.mellifera)*	Apidaecin-qR	GCAGGTCGAGTAGGCGGATCT		
qPCR	*ß-actin*	*ß-actin*-qF	AGGAATGGAAGCTTGCGGTA	[68]	181
	*(A.mellifera)*	*ß-actin*-qR	AATTTTCATGGTGGATGGTGC		

Underlining and lowercase letters shows the T7 promoter sequence.

## Data Availability

Not applicable.
